# Supplementary material for: Choose Your Own Adventure: Using Twine for Gamified Interactive Learning in Veterinary Anaesthesia
Source: Vet Sci. 2025 Feb 11;12(2):156. doi: 10.3390/vetsci12020156 (PMC11860683; doi:10.3390/vetsci12020156)
Supplement: Supplementary file 1 [file vetsci-12-00156-s001.zip › vetsci-3390231-supplementary.pdf]

Casos clínicos interactivos

Quiero contar contigo para mejorar nuestra experiencia de aprendizaje. ¿Te animas a compartir tu opinión sobre la actividad de casos clínicos en Twine? Tu opinión es súper importante para nosotros. Ayúdanos a entender qué te ha parecido, qué te ha gustado más y en qué podríamos mejorar. ¡Tu voz cuenta!

¡Gracias por tu colaboración!
1. ¿En qué asignatura has hecho esta actividad?
Respuesta necesaria. Opción única.
- ICV
- CAC I
- CAC II

2. Sexo
Respuesta necesaria. Opción única.
- masculino
- femenino
- prefiero no decirlo

3. ¿A qué rama de la veterinaria te gustaría dedicarte cuando acabes la carrera?
Respuesta necesaria. Opción única.

- Clinica de pequeños animales
- Clínica de grandes animales
- Producción animal
- Ciencia y tecnología de los alimentos
- Otra

4. ¿Te gusta la anestesia? Respuesta necesaria. Likert.

5. ¿Consideras que este taller te ha ayudado a tu formación en anestesia veterinaria? Respuesta necesaria. Likert.

6. ¿Consideras que este método de usar casos interactivos es útil para mejorar tus conocimientos en anestesia? Respuesta necesaria. Likert.

7. ¿Encontraste fácil de usar la plataforma Twine para interactuar con los casos clínicos? Respuesta necesaria. Likert.

8. En general, ¿cómo calificarías tu experiencia en el taller? Respuesta necesaria. Likert.

9. ¿Tienes algún comentario adicional sobre el taller que te gustaría compartir? ¿Qué es lo que más te ha gustado más? ¿Qué podemos hacer para mejorar la actividad? Respuesta opcional. Abierta

10. ¿Cómo calificarías la claridad de las instrucciones? ¿Encontraste útil la estructura de los casos clínicos en Twine? Respuesta opcional. Abierta

11. ¿Hay áreas que podrían ser más efectivas o más interesantes? ¿Qué cambios sugerirías? Respuesta opcional. Abierta

12. ¿Tienes algún comentario adicional que te gustaría compartir sobre la actividad?
Respuesta opcional. Abierta
